# Toward Mapping an NGSI-LD Context Model on RDF Graph Approaches: A Comparison Study

**DOI:** 10.3390/s22134798

**Published:** 2022-06-24

**Authors:** Ahmed Abid, Jieun Lee, Franck Le Gall, JaeSeung Song

**Affiliations:** 1 EGM, Sophia Antipolis, 06560 Valbonne, France; franck.le-gall@egm.io; 2 Department of Computer Security and Convergence Engineering for Intelligent Drones, Sejong University, Gwangjin-gu, Seoul 05006, Korea; love9ly@sju.ac.kr (J.L.); jssong@sejong.ac.kr (J.S.)

**Keywords:** NGSI-LD, semantics, RDF graph mapping

## Abstract

A considerable number of Internet of Things deployments are isolated from specific solutions, from devices to data platforms. Standardized data models were proposed to overcome the interoperability gap between these deployments. Next generation service interfaces-linked data (NGSI-LD) is one of the proposed platforms that exploits linked data and proposes an information model and an application programming interface (API) for easy use and standard management of context information. The NGSI-LD information model is based on JSON for Linked Data (JSON-LD) as a serialization format for context information. This efficiently exploits the potential of semantics and linked open data. However, the NGSI-LD graph API and query language are still theoretically defined and limited to some preliminary works. Consequently, current NGSI-LD implementations are mainly based on traditional databases, where the JSON-LD serialization is supported but not exploited owing to the difficulties in defining and implementing new NGSI-LD based Graph APIs. One of the basic solutions is the use of an RDF store for NGSI-LD payloads because these types of databases are well defined and maintained and will not need any added effort for JSON-LD based payloads. However, the main complication at this level is the use of reification to annotate relationships. This study focused on both aspects of exploiting the semantics of NGSI-LD by proposing standardized mapping mechanisms to RDF graphs without reifying JSON-LD payloads and with respect to the NGSI-LD context model and API. Our main proposals highlight that exploiting the RDF store for processing NGSI-LD data semantically is feasible and uncomplicated. We illustrated the proposed mapping approaches with real use-case examples and a possible exploitation of semantic approaches.

## 1. Introduction

Since their inception, Internet of Things (IoT) networks have continued to grow successfully within the scientific and industrial communities. Because of its various advantages, this technology is considered a key player in current network architectures, opening up several technical challenges and immense application possibilities. The IoT is an emerging area that not only requires development of infrastructure but also deployment of new services capable of supporting multiple, scalable, and interoperable (multi-domain) services. Over these IoT environments, data present the bridge that connects cyber and physical worlds. Thus, to reach its full potential, produced data must be dynamically and automatically processed and exposed for use in interoperable ways by various applications and services such as decision making, machine learning, and real time applications.

In IoT environments, data is characterized by its heterogeneity because of the variety of data sources such as satellites, sensors, global positioning system (GPS), and their various formats ways of produced data due to the increasing number of organizations that produce these devices. Several other problems related to IoT resources such as deployment scale, constrained devices, constrained resources, and intermittent loss of connection endanger the quality of produced data. These issues certainly affect the quality of services promised by the IoT systems.

To overcome these issues, data validation process has become the focus of researchers, business, and most of the standards organizations in the context of IoT over the past years. The main objective is to develop standardized and autonomous tools for processing the increasing amount of heterogeneous raw data over IoT platforms.

In this context and following the increasing industry adoption of semantic Web technologies (https://www.w3.org/standards/semanticweb/, visited on 21 June 2022), several ontologies and data models have been defined and standardized, serving as a reference model for IoT data and covering different aspects in the IoT layers (knowledge, information, data and physical layers) [1]. The smart appliances reference (https://ontology.tno.nl/saref/, visited on 21 June 2022) (SAREF) ontology [2] has been developed and standardized by the European Commission in close cooperation with European Telecommunications Standards Institute (ETSI) to provide a modular and domain-independent semantic layer for smart appliances. The semantic sensor network (SSN) [3] and sensor, observation, sample, and actuator (SOSA) are a set of ontologies that describe sensors, actuators, and samplers as well as their observations, actuation, and sampling activities (https://www.w3.org/TR/vocab-ssn/, visited on 21 June 2022), which have been published as both a W3C recommendation and as an OGC [4] implementation standard. The ontology set adopts a modular architecture with SOSA as a self-contained core with SSN as an extension to add expressivity and breadth supporting a range of sensing- and actuating-based cases, such as scientific monitoring, industrial infrastructures, and IoT. The Web of Thing [5] (WoT) ontology is defined within W3C’s WoT working group as a thing-centric formal model and a common representation for WoT. A thing is an abstraction of a physical or virtual entity which interacts and participates in the WoT, and a thing describes the metadata and interfaces of things.

Other initiatives have focused on proposing completly interoperable IoT platforms covering the major steps of the IoT data life cycle. oneM2M [6] is a global partnership project that aims to create a global technical standard for interoperability concerning the architecture, application programming interface (API) specifications, security, and enrolment solutions for machine-to-machine and IoT technologies based on requirements contributed by its members. Fiware (https://www.fiware.org/), visited on 21 June 2022 is an open-source platform offering a set of generic enablers, which encompass a framework that allows the development of smart applications that rely on smart services and data management components. Fiware generic enablers are grouped into several categories. One of them is related to context management and involves software components for smart city services which use IoT and its related metadata.

ETSI (https://www.etsi.org, visited on 21 June 2022) realized the next generation service interfaces-linked data (NGSI-LD) standard for the Fiware community platform. The NGSI-LD standard is the evolution of the NGSI v2 specification (https://fiware.github.io/specifications/ngsiv2/stable/, visited on 21 June 2022) that supports linked data by adopting the JSON-LD (https://json-ld.org/, visited on 21 June 2022) serialization. NGSI-LD standard proposes an information model and an API to produce, consume, and subscribe to context information. The current implementations of the NGSI-LD are provided as context brokers that support the NGSI-LD data model and the APIs functionalities.

The NGSI-LD APIs functionalities are fully based on the data received from the context producers. Thus, the data exchanged in the NGSI-LD context broker depends totally on the information sent from the context producers. In the IoT context and with the adoption of new processes to fully automate the sending of data from IoT devices, the API remains limited in checking the consistency of the received data, although the data in NGSI-LD is serialized in a linked data format. The NGSI-LD data model is assisting producers and consumers in structuring their data and thus finding a way for exchanging them even when they are not fully aligned with the data model that the API accepts. Data quality evaluation and verification and semantic validation and reasoning processes are clearly missing.

Following the semantic Web technologies, the Resource Description Framework (https://www.w3.org/RDF/, visited on 21 June 2022) (RDF) has been designed to share and manage large collections of linked data. The performance and flexibility of this model lies in its use of a simple unified data representation, namely triples. The exploitation of these potentials is limited by the fact that the serialization of NGSI-LD payloads in RDF-based semantic databases (triple stores) is a complex task, especially when annotating relationships [7]. For this purpose Most NGSI-LD implementation have only considered syntactical databases where the linked data aspect of JSON-LD only serves as a definition of the NGSI-LD attributes in an expanded format.

We consider this study as the earliest to propose efficient mapping approaches from NGSI-LD property graph to RDF graphs that enhance and exploit the semantic description of the NGSI-LD payloads. Based on these approaches, NGSI-LD context broker may use on of the existing triple stores instead of implementing a graph API from scratch. The proposed mapping approaches are fully aligned with the NGSI-LD data model. Based on RDF graphs, semantic tools and specifications may be applied. In this study we also show the benefits of semantic enhancement via examples of semantic data validation and semantic reasoning, such as entity multi-typing (sub-classing), that may be processed on the generated property graph. Within the proposed approach, the NGSI-LD data model and API remain unchanged and supported, and the mapping approach and the validation processes and design are integrated into the context broker while supporting the NGSI-LD specifications. The proposed approaches in this paper are illustrated via use case examples. Semantic extension, data validation, and reasoning were tested, validated and adopted on a NGSI-LD compliant broker.

The reminder of this paper is organized as follows. Section 2 introduces the basics of the NGSI-LD data model and API. Two motivating scenarios are presented and illustrated with NGSI-LD serialization in Section 3. In Section 4, mapping strategy approaches are detailed and compared, then applied on use-cases, and finally compared and criticized. Section 5 presents the main benefits of supporting such mappings by exploiting semantic technologies. Section 6 depicts main existing approaches working on mapping property graphs to RDF ones and existing approaches that support semantic enhancement over NGSI-LD extension property graphs. Finally, Section 7 concludes the paper.

## 2. NGSI-LD Context Model and API

The Industry Specification Group for Cross-cutting Context Information Management (ISG CIM) was formed in ETSI in 2017 to improve interoperability and reduce deployment problems for smart city services that use IoT and/or metadata. The goal of the ISG CIM is to issue technical specifications to enable multiple organizations to develop interoperable software implementations of a cross-cutting context information management layer, which enables applications to discover, access, update, and manage context information from different sources, as well as publish it through interoperable data publication platforms. The NGSI-LD data model is a work item dedicated to designing a data-centric ontology that supports data management by its implemented APIs. Details of the NGSI-LD data model and API are presented in the following sections.

### 2.1. NGSI-LD Data Model

**Figure 1:**
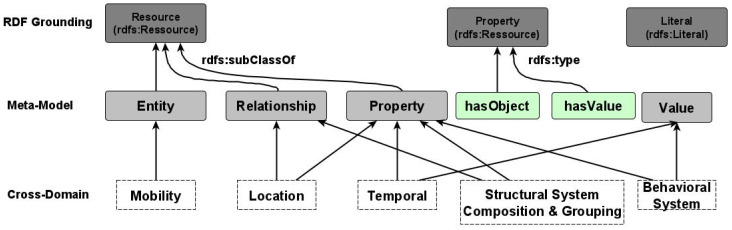
NGSI-LD Data Model Layers.

As shown in Figure 1, the NGSI-LD data model is an extension of the RDF standards to capture high-level relations between entities and properties.

The Meta-Model primarily includes:

- Entity, which is the core concept in NGSI-LD. The NGSI-LD entity is an informational representation that is supposed to exist physically or conceptually. NGSI-LD is an entity model;
- Relationship, which captures possible links between a subject which maybe an entity, a property, or another relationship on the one hand, and an object, which is an entity, on the other hand.
- Property, is a description instance, which associates a main characteristic to either an entity, a relationship or another property.

The Meta-Model also includes the hasObject and hasValue to affect literal values to relationships and properties. At a cross-domain level, extensions are made to further describe the general concepts at the meta-model level, which include other concepts in various sub-domains:

- Mobility defines the stationary, movable, or mobile characteristics of an entity.
- Location differentiates and provides concepts to model the coordination-based, set-based, or graph-based location.
- Temporal specification includes property and values for temporal property definitions.
- System composition and grouping provides a way to model a system of systems in which smaller systems are integrated to form a complex system following specific patterns.
- Behavioral system includes properties and values to describe system state, measurement, and reliability.

An entity is composed of an id, a type, a set of properties, a set of relationships, and a @context field following the JSON-LD specification. Properties and relationships may also include nested sets of properties and relationships (such as property of property, relationship of property, and property of relationship). @context contains the definition of all attributes, via including their Uniform Resource Identifiers (URIs).

### 2.2. NGSI-LD API

The NGSI-LD API relies on the data model presented in the previous section. It provides a set of operations on entities covering entity creation, update, deletion, retrieval, and subscription. The API also proposes operations that include registry, batch, and temporal operations.

In the current version of the NGSI-LD specification, the API does not exploit the URIs of attributes defined in the @context field. In fact, the @context field is mandatory in each NGSI-LD entity, but the operations offered by NGSI-LD API focus more on the syntactic side of entity attributes such as temporal values and partially ignore the semantic side. This certainly does not affect the efficiency of the API, but processes such as data validation and semantic reasoning are not fully exploited despite the existence of the semantic description (URIs).

## 3. Use-Cases

To present and detail RDF mapping approaches, we present in this sections two real use-case examples modelled in NGSI-LD these examples follow recommendations of [8].

### 3.1. Water Domain Use Case Application

We consider a use case from the undergoing modelization work of the water network management (https://github.com/smart-data-models/dataModel.WaterNetworkManagement, visited on 21 June 2022).

The use case consists of a set of devices to measure water parameters in a city water network. The parameters are chlorine and water levels in a tank.

**Figure 2:**
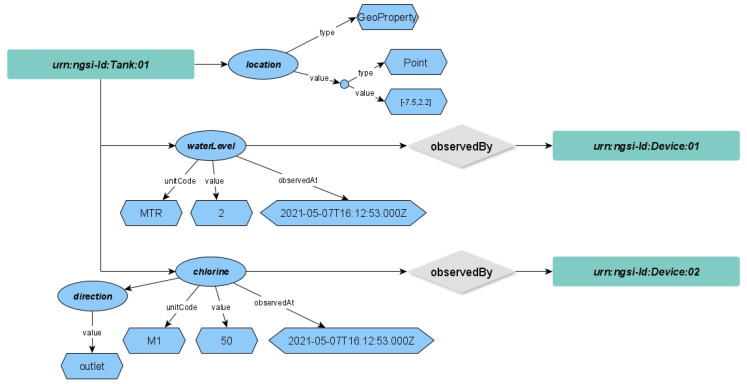
Tank NGSI-LD Entity Data Model.

The tank will follow the data model proposed for a tank in the water management network. In this use case, three new properties will be added: waterLevel and chlorine. A graphical representation of this data model is depicted in Figure 2.

The JSON-LD serialization of the tank entity is depicted in Listing 1.

**Listing 1.** A Json-LD serialization of Tank Entity.

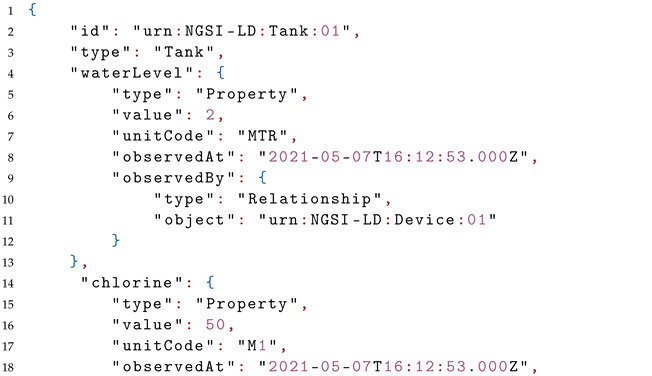


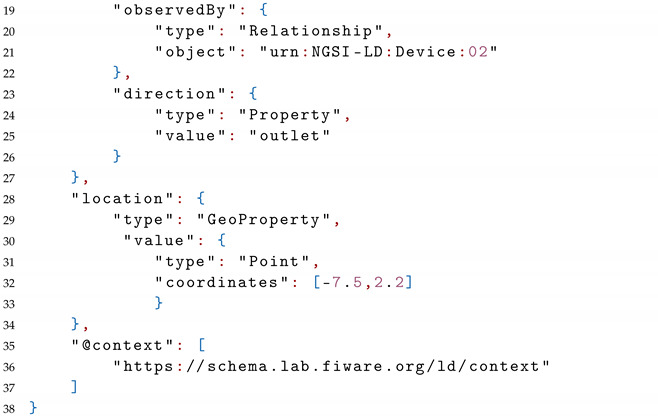



The @context contains the definition of all referred attributes of the tank entity.

### 3.2. Infection Domain Use Case Application

In this section, we consider a use case about the COVID-19 infection situation in South Korea. In South Korea, Korea Centers for Disease Control & Prevention (KCDC) announces the information related to COVID-19. The reports provided by the KCDC and the Korean local government include simple information about the infected person, such as gender and age, information about the place the infected person visited, and information about the infected group. Following the NGSI-LD specification, we have designed NGSI-LD entities based on the report materials.

#### Infection Domain NGSI-LD Model

**Figure 3:**
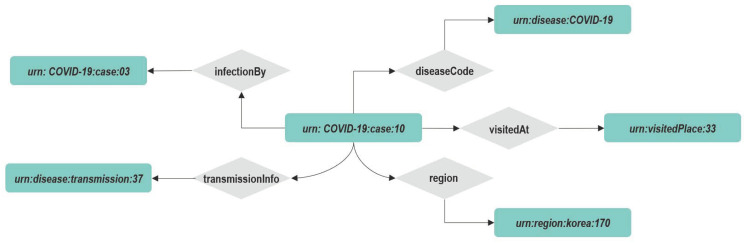
Infection NGSI-LD Entity Relationship Data Model.

The NGSI-LD data model we designed includes infection case, disease, disease transmission, region, and visited place as NGSI-LD entities. The relationships between the NGSI-LD entity will be modelled as “infectionBy”, “visitedAt”, “transmissionInfo”, “diseaseCode”, and “region”. The NGSI-LD entity relationship graphical model of these use cases is depicted in Figure 3.

**Figure 4:**
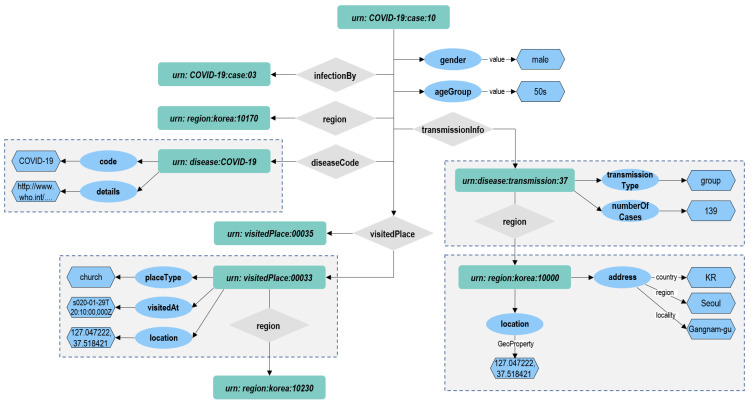
Infection NGSI-LD Entity Data Model.

A graphical representation of a portion of this data model is shown in Figure 4.

The JSON-LD (partial) serialization of the infection case entity is depicted in Listing 2.

**Listing 2.** A partial JSON-LD Serialization of Infection Case Entity.

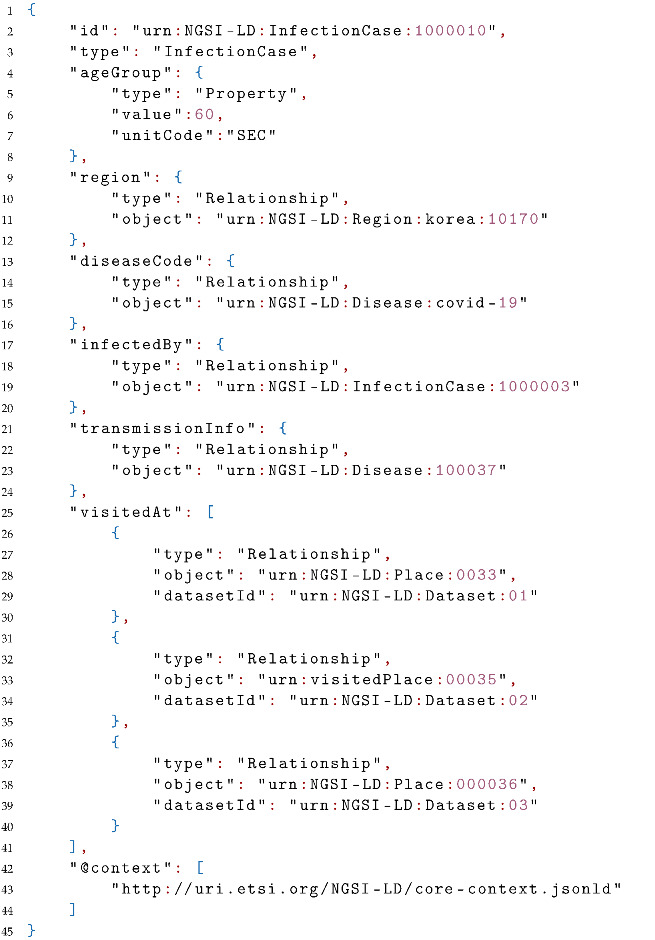



## 4. NGSI-LD to RDF Graph Mapping Strategies

As JSON-LD is one of the possible serialization formats proposed for linked data, the mapping seems to be a simple task, especially when using a triple store, and thus exploits entity URIs in semantic reasoning. In the following sections, we present the main issues encountered when mapping NGSI-LD entities to RDF graphs as motivations to propose a new NGSI-LD mapping approach to property graphs.

### 4.1. NGSI-LD to RDF Graphs via Reification

As NGSI-LD entities are serialized in JSON-LD, it is possible to import these entities directly to triple stores. However, this process seems to be a very complex because RDF reification (https://www.w3.org/DesignIssues/Reify.html, visited on 21 June 2022) is the default way to model property graphs, by allowing the attachment of properties to relationships and supporting any meta-data on them. The process of reification is based on automatically creating RDF blank nodes between different RDF classes. These blank nodes are exploited by attaching all meta-data to them.

**Figure 5:**
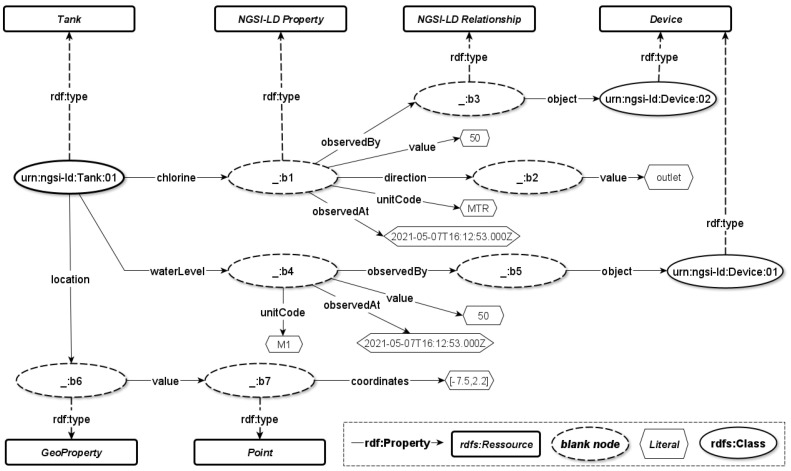
RDF Graph for Tank NGSI-LD Entity.

Figure 5 graphically depicts a mapping of the NGSI-LD tank entity using the RDF mapping. This figure shows that several blank nodes were added explicitly during the mapping process.

### 4.2. NGSI-LD to Named RDF Graphs

The named property graph approach also follows the basics of semantic Web specifications. It is mainly based a triplet format, where a triplet consists of a subject (node), an object (node), and a relation (vertex) between the subject and the object. The main mapping rules of this approach consist of mapping an NGSI-LD entity as follows:

- Each NGSI-LD entity is mapped as a subject node. The type of this node corresponds to the type of the entity. Entity Id, temporal attributes, location, and context are mapped as literal properties of the node.
- Each NGSI-LD property is mapped as an property-type object node. The vertex relating the entity node to its property is named “has_value”. The property name, value, temporal attributes, and unit code are mapped to literal properties of the node. In the case of a property of property in NGSI-LD, each property will be defined as separated node and a new “has_value” relation will be added between them.
- Each NGSI-LD relationship is mapped as an relationship-type object node. Temporal attributes are mapped to literal properties of the node. The vertex relating the entity node to its relationship is named “has_object”. The relationship node is related to the subject entity node via the named vertexes “has_object” and the name of the relationship. Sub-relationships and sub-properties of a relationship follow the same principles.

#### Application on Water Use Case

**Figure 6:**
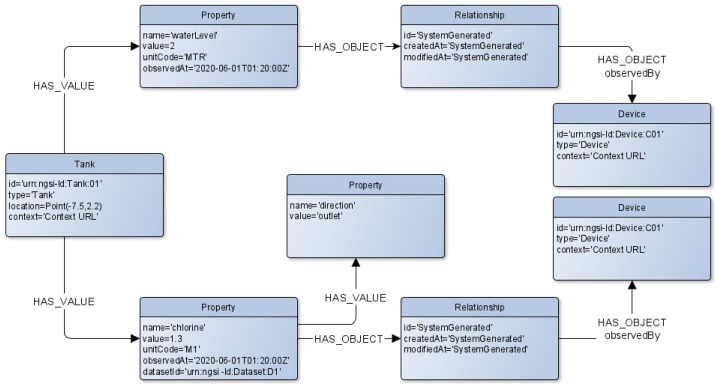
Mapping Tank Entity to a Named Property Graph.

The application of the named property graph approach to the water use case in Section 3 is depicted in Figure 6. Each NGSI-LD entity, property, and relationship are mapped to a separate node, where the type of the entity “Tank” is the type of the nodes. The tank node is characterized by the following attributes: id, type, and context. It is also connected to a property-type node via the edge named “HAS_VALUE”. The property node models the NGSI-LD property “chlorine”, which is characterized by the following attributes: name, value, unitCode, datasetId, and observedAt. Its node is related to two other nodes of property and relationship types via the edges “HAS_VALUE” and “HAS_OBJECT”, respectively. This property node presents the sub-property “direction”. The relationship node relates the chlorine property node to the device entity node.

### 4.3. NGSI-LD to Weighted RDF Graphs

The focus of the weighted RDF Graph approach is on converting it to a property graph and making it available in the Arango graph database. As a result, the expressive AQL graph query language can be used on the NGSI-LD, which provides the basis for extracting knowledge and detecting situations.

A graph database follows the basics of the property graph model specifications. It consists of a vertex (node), an edge (relationship), and a property. Both vertices and edges may have properties and the edge organizes vertices. Two vertices are connected by the an edge. The main rules of the proposed mapping approach B consist of mapping NGSI-LD entity elements as follows:

- Each NGSI-LD entity and NGSI-LD property are mapped as a vertex, and the NGSI-LD relationship is mapped as an edge. The reason for mapping the NGSI-LD property to a vertex instead of an edge is that NGSI-LD properties can have metadata in form of NGSI-LD properties. Because of this mapping, NGSI-LD properties of NGSI-LD properties are also mapped to the vertex.
- Non-reified properties of NGSI-LD entities like “createdAt” and “modifiedAt” are mapped to the property of a vertex or an edge.
- The NGSI-LD property is mapped as a new vertex and connected with the NGSI-LD entity vertices through the edges. This mapping is the same for NGSI-LD property of NGSI-LD property.
- The id of the NGSI-LD entity is mapped to a vertex id (key) and the type of the NGSI-LD entity is mapped to the property of vertex. The attribute name and type of the NGSI-LD relationship are mapped to the property of vertex or edge.
- The NGSI-LD entity is assigned to the vertex corresponding to “from”, and the vertex corresponding to the object of the NGSI-LD relationship becomes the vertex corresponding to “to” so that the NGSI-LD relationship is mapped to the edge.

#### 4.3.1. Application on Water Use Case

**Figure 7:**
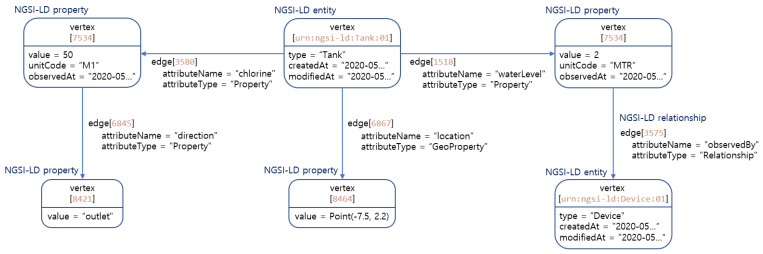
Mapping Tank Entity to Graph DB.

The application of the weighted RDF Graph approach to the water use case in Section 3.1 is depicted in Figure 7. Each NGSI-LD entity and property are mapped to a separate vertex. The key of the tank vertex is the id of NGSI-LD entity, and the tank vertex is characterized by the following attributes: type, createdAt, and modifiedAt. Every NGSI-LD property vertex is connected to the tank vertex via the edge. The property vertex models the NGSI-LD property “chlorine” and “waterLevel”, which are characterized by value, unitCode, and observedAt. The property vertex models the NGSI-LD geoproperty “location”, which is characterized by the point-type value. Its node is related to two other nodes which have the types property and relationship via the edge for “NGSI-LD Property” and “NGSI-LD Relationship” respectively. This property vertex presents the sub-property “direction”. The relationship edge is related to the property vertex waterLevel and to the device entity vertex.

#### 4.3.2. Application on Infection Domain

**Figure 8:**
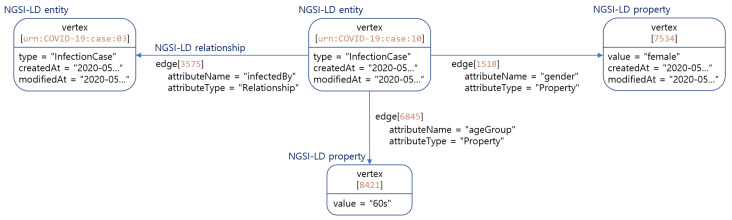
Mapping Infection Entity to Graph DB.

The application of the Graph DB approach to the water use case in Section 3.2 is depicted in Figure 8. Each NGSI-LD entity and property are mapped to a separate vertex. The key of the COVID-19 case vertex is the id of NGSI-LD entity, and the COVID-19 case vertex is characterized by the following attributes: type, createdAt, and modifiedAt. Every NGSI-LD property vertex is connected to the COVID-19 case vertex via the edge. The property vertex models the NGSI-LD property “gender” and “ageGroup”, which are characterized by value, createdAt, modifiedAt, and observedAt. Its node is related to two other nodes which have the types property and relationship via the edge for “NGSI-LD Property“ and “NGSI-LD Relationship”, respectively. The relationship edge is related to the entity vertex COVID-19 case to another COVID-19 case entity vertex.

## 5. Validation and Semantic Applications Examples

Semantic integration over NGSI-LD context broker enhances the integration of semantic data validation processes through consistency checking over RDF schemes. Semantic inference and reasoning mechanisms can also be exploited to enrich and annotate existing data through graph-traversing and matching queries across instances, hyper-structural constructs, types, and ontologies.

### 5.1. NGSI-LD to Named Property Graph Approach

The proposed approach is a fully adapted mapping by context broker Stellio (https://www.stellio.io/, visited on 21 June 2022). Entities, properties, and relationships in Stellio are stored as a set of linked nodes via labelled edges.

Any updates on attributes of this entity will update the values of the nodes and produces a new storing event in time series databases for the former values. In such a process, the context is stored in a graph database along with the latest values of properties and relationships. The former values of each updated attribute are transmitted to an efficient time series database.

Queries over Stellio are then processed according to their nature. Queries about current state of entities are processed in the graph database as they are stored there. Queries about the temporal evolution of an entity (historical data) are processed in the time series database as they are stored there.

Stellio exploits the potential of the Neo4j (https://neo4j.com/, visited on 21 June 2022) database. Developed in Java, Neo4j is one of the most popular graph database management systems. Neo4j is easily accessible from external software written in different languages via the expressive and efficient cypher query language developed initially for Neo4j needs. Neo4j also offers a collection of the latest innovations in graph technology that exploits stored data, such as the NeoSemantics (https://neo4j.com/labs/neosemantics/, visited on 21 June 2022) library which offers various tools to manage linked data and supporting ontologies and semantic inferences. This library is integrated in Stellio to exploit the potential of references and semantic definitions from a context field attached to NGSI-LD entities.

Application on water use case

Let us suppose that the tank entity of listening Figure 2 is created in the context broker via the NGSI-LD API. The correspondent named property graph as described in Figure 6, will be available on its internal graph database, Neo4j. Hereinafter, we will show some possible applicable features on such a knowledge base.

Multi-typing over sub-classing

On Neo4j, let us import the following snippet (Listing 3) of ontology defining a tank from the Saref for water (https://saref.etsi.org/saref4watr/, visited on 21 June 2022) ontology.

**Listing 3.** Tank Definition serialized in N3.

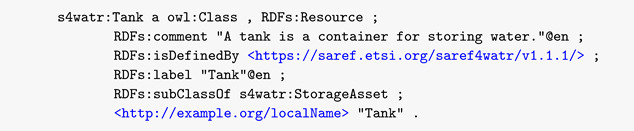



Integrating this knowledge will allow discovering tank entities not only with their syntactic type “Tank” but also with the type of their super-class storage asset because a tank is also defined as RDFs:subClassOf StorageAsset. Via the API, the response of searching entities of type “StorageAsset” will include entities of type “Tank”.

schemes validation

Current NGSI-LD APIs validate only the NGSI-LD data model conformity of incoming data. Through RDFS schemes and knowledge based on graph database, it is possible to impose semantic constraints when manipulating NGSI-LD data.

It may also be possible for RDFS schemes and ontologies to make graph-based searches by validating data against RDFS schemes.

SHACL Validation

SHACL (https://www.w3.org/TR/shacl/, visited on 21 June 2022) is one of the possible features for validating data graph-based data against a set of conditions and to infer new triples based on existing ones. The integration of this process of validation is through the graph database.

Listing 4 shows an example of SHACL validation applied on StorageAsset classes. This query checks for all storage asset classes and sub-classes if a property “HAS_OBJECT” points to a class of type “https://ontology.eglobalmark.com/egm#managedBy” (visited on 21 June 2022). From the NGSI-LD perspective, this validation query will check if all entities of type storage asset and their sub-class types have a “managedBy” relationship.

**Listing 4.** SHACL validation Example.

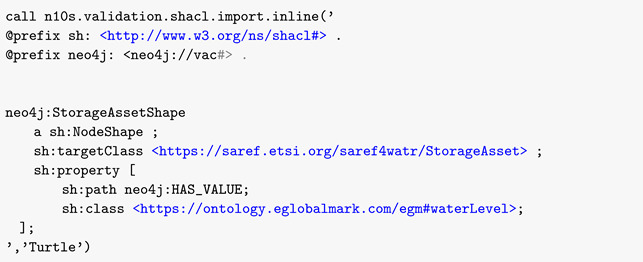



### 5.2. NGSI-LD to Graph DB

Unlike Stellio, the proposed approach is not a Neo4j database, but a mapping fully adapted by an NGSI-LD context broker that leverages the potential of Arango DB. Developed in C++, Arango DB is a multi-database that supports not only graphs but also documents and key-value data models. Arango DB is easily accessible from all data models via the Arango DB query language (AQL) which is one SQL-like query language.

Arango DB can accommodate the complex NGSI-LD data model. For example, when an NGSI-LD entity is substituted for a vertex and an NGSI-LD relationship is substituted for an edge, semantic data with complex relationships such as relationship of relationship or relationship of property can be expressed.

Application on Infection domain

Let us suppose that the tank entity of Listing 2 is created in the context broker via the NGSI-LD API. The correspondent named property graph, as described in Figure 8, will be available on its internal graph database, Arango DB. We will show some possible applicable features on such a knowledge base.

Scaling with domains

Despite large data volumes because of thousands or tens of thousands of infections per day, a single domain can handle large data sets and scale vertically without problems. However, because of the semantic property of being able to connect between different domains through the mashup technology, the possibility of extending the NGSI-LD domain should be considered as the expansion of ontology is possible.

Imagine doing a deeper graph traversal with highly interconnected communities through a horizontal scaling to more domains such as place and time information. Because such a large amount of data will not fit in a single system, let us import a large data set of infected domains in server A on Arango DB. Further, the data sets on the place and time information to be connected with the infection cases are imported into servers B and C, respectively. Arango DB enables semantic mashup of large data sets between domains by providing an edge on path and network hops between servers and speeds up graph traversal.

## 6. Related Work

The NGSI-LD data model is mainly based on property graph model. Pushing and requesting NGSI-LD property graphs into graph database seems to be a time consuming task due to RDF reification issues (see Section 4.1).

In [9] the authors proposed an evolution framework based on four layers by combining the property graph principles and RDF-based semantic modeling. This work was the first trial around extending the NGSI-LD to bring to it a graph-based semantics.

Authors in [10] have tried to exploit semantic technologies by pushing NGSI-LD property graph to graph database and then presented efficient examples of Sparql (https://www.w3.org/TR/RDF-sparql-query/, visited on 21 June 2022) queries based on the uploaded via an Event Processing Architecture. However, the performance of this approach is not guaranteed due to reification issues.

Following the main contribution of this paper, the rest of our bibliographical study will focus on a review of exiting property Graphs to RDF mapping approaches, especially approaches that consider reification issues.

In [11] the authors propose three data models to support property graphs as RDF in the Oracle database. These models are presented based on a named graph, subproperty, and extended reification. This approach shows that mapped named graphs achieve a shortened response time in SPARQL queries. A similar approach was presented in [12]. The approach maps the property graph model, hypergraphs, and other data structures to RDF reification. This approach also clarifies a means to query such data using SPARQL. Unfortunately, unlike graph queries, when executing graph traverses with SPARQL queries, both solutions increase query execution time steeply as the path length increases due to exponential complexity. These solutions are particularly vulnerable to data models with complex relationships, such as NGSI-LD.

The RDF data model can be simply mapped to property graph model [13,14,15,16]. This means that the NGSI-LD data model based on RDF grounding is also possible mapped to a property graph model with suitable mapping strategies. Especially the concept of a named property graph, one of the NGSI-LD to RDF graph mapping strategies we proposed, has long been presented in previous research [17]. The NPG model is based on a general property graph with a non-empty set of named nodes. The authors provide NPG and algorithms for mapping their proposal into existing databases. Unfortunately, NPG needs separate query language to support the mapping algorithms.

These mapping approaches seem to be efficient approaches may be applied in the context of mapping generic property graphs to RDF ones. However, the main issue with these approaches is the specificity of the NGSI-LD data model (see Section 2) and API. In fact, to exploit semantics over NGSI-LD based context brokers, the mapping principles should deal with the entity relationship data model of NGSI-LD and should support NGSI-LD property and relationship specifications. The proposed approaches in this paper consider only mapping the NGSI-LD property graph and intend to bring graph-based semantics to the NGSI-LD context broker.

## 7. Conclusions

Integrating a graph API within NGSI-LD context broker seems to be an interesting and promising feature because of the newly added capabilities of exploiting semantic processes. We present the mapping strategies which allow mapping the NGSI-LD payloads into RDF graphs without using reification mechanisms and by avoiding a new implementation of graph NGSI-LD dedicated API. Our proposed mapping strategies provide fully compliant approach with the NGSI-LD context broker data model and API. We also introduce semantic processes that could be a very simple task such as sub-classing reasoning and semantic data validation is exploited directly by existing NGSI-LD context brokers. We consider our approach one of the first works around bringing semantics to NGSI-LD brokers. This proposal can easily bring and enhance semantics to NGSI-LD brokers by only adopting these mapping processes. These processes are approved and adopted by the NGSI-LD compliant context broker Stellio.

Currently, over the Stellio context broker, the semantic processes are executed in background mode, and we are getting daily reports about the data quality over the context broker according to knowledge rules. We are working on extending the execution of the semantic processes by defining new NGSI-LD compliant ways to upload the knowledge, exploit and execute semantic processes then expose these results. In future work, we will focus on exploiting more semantic technologies over NGSI-LD data imported from RDF stores. Another challenge is to use open linked data, and schemes search over NGSI-LD based RDF schemes patterns.

## Data Availability

Not applicable.
